# Supplementary material for: Memory Consolidation Is Linked to Spindle-Mediated Information Processing during Sleep
Source: Curr Biol. 2018 Mar 19;28(6):948–954.e4. doi: 10.1016/j.cub.2018.01.087 (PMC5863764; doi:10.1016/j.cub.2018.01.087)
Supplement: Document S1. Figures S1–S4 and Tables S1–S4 [file mmc1.pdf]

Current Biology, Volume *28*

Supplemental Information

Memory Consolidation Is Linked to Spindle-Mediated Information Processing during Sleep

Scott A. Cairney, Anna á Váli Guttesen, Nicole El Marj, and Bernhard P. Staresina

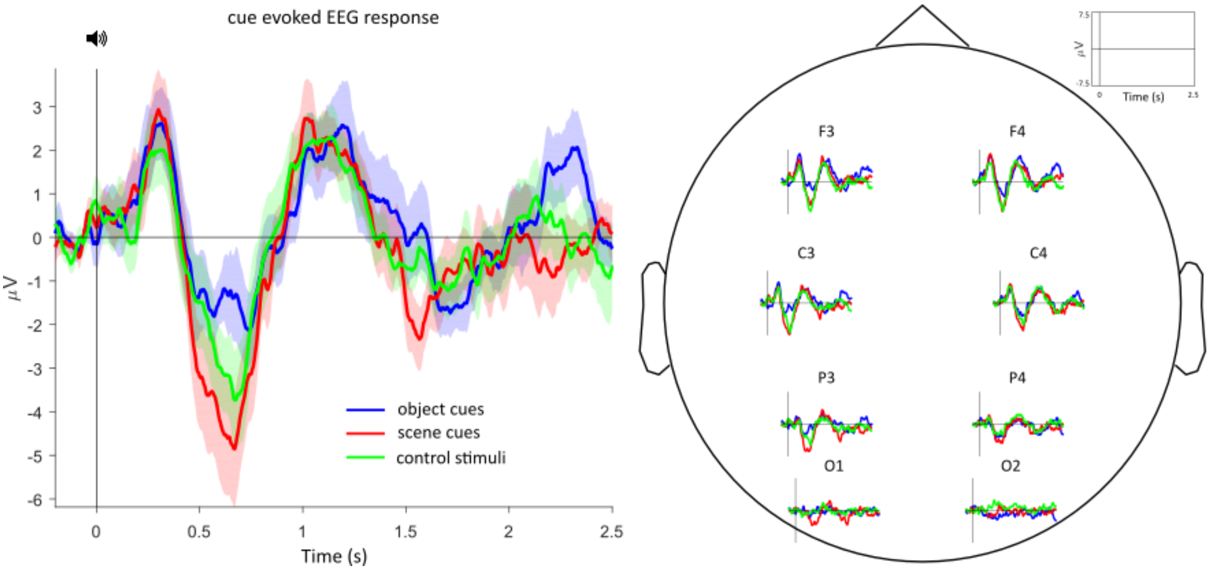

Figure S1: Event Related Potentials (ERPs) in Response to Auditory Cues, Related to Figure 2B *Left*: Traces show mean ± SEM across participants, collapsed across all electrodes. *Right*: Condition means across participants, shown for each electrode separately. A repeated-measures ANOVA revealed no significant condition effects comparing object cues, scene cues and control stimuli after controlling for multiple comparisons across time and electrodes.

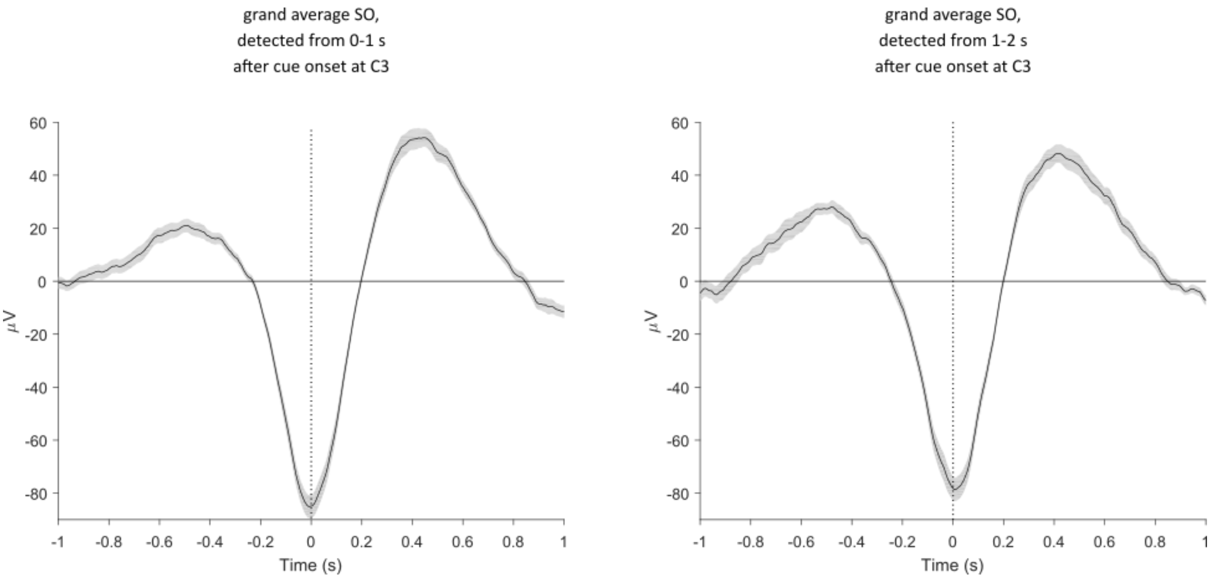

Figure S2: Grand Average Slow Oscillations, Related to Figures 2 and 3. Algorithmically detected slow oscillations (see STAR Methods) whose maximum trough ('down state') fell between 0-1 s (*left*) or 1-2 s (*right*) post cue onset, shown for electrode C3. Traces show grand average ± SEM across participants. ± 1 s raw data segments around the SO trough (time 0) were extracted to accommodate the maximum SO duration of 2 s. Note that both early and late SOs surpass the standard peak-to-peak amplitude criterion of 75μV [S2].

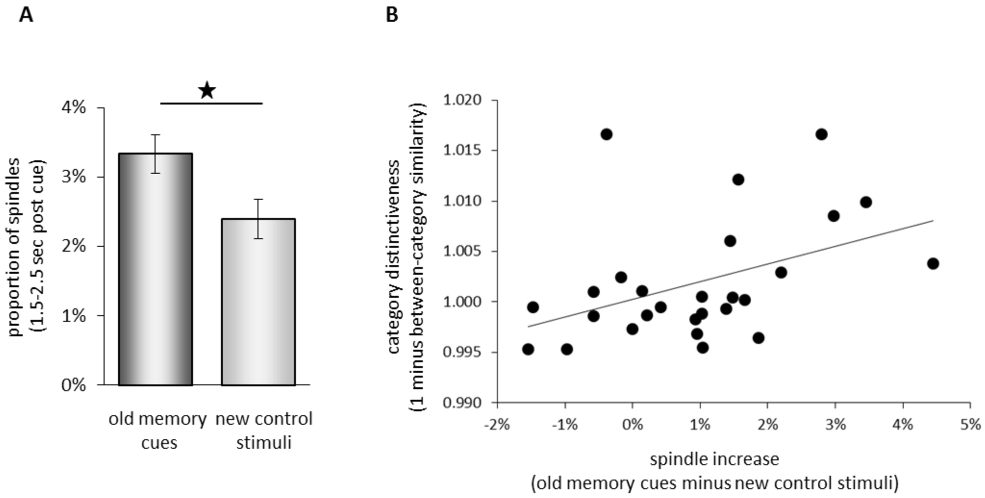

__**Figure S3: Discrete Spindle Events from 1.5-2.5 s post cue onset, Related to Figure 3**__ **A.** Relative count of algorithmically detected fast spindles (13-16 Hz) for old memory cues and new control stimuli, averaged across left hemisphere electrodes. Star indicates significant difference ($t(26)=3.33$, $P=.003$) **B.** Across-participant correlation between (i) relative spindle increase for old cues vs. control stimuli (cf. panel **A**) and (ii) category distinctiveness of object- and scene-related cues (Spearman's rho=.47, $P=.014$).

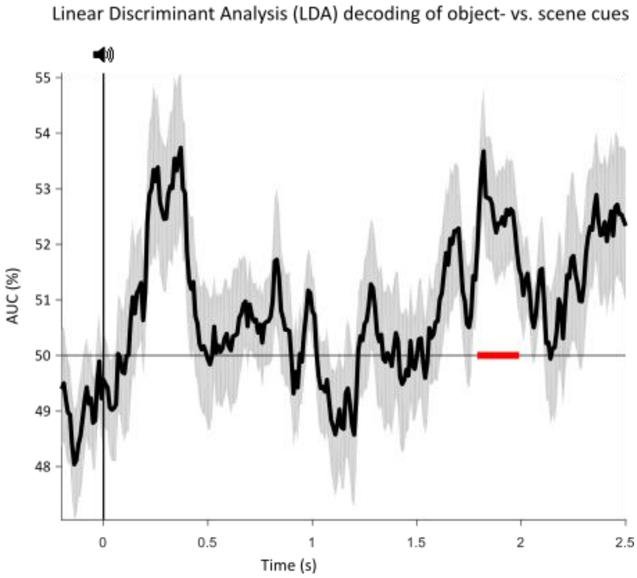

__**Figure S4: Linear Discriminant Analysis (LDA) decoding of object vs. scene cues, related to Figure 4A**__ Classification was performed analogously to the RSA analysis described in the main text. At each timepoint (-.2 s to 2.5 s relative to cue onset, 10 ms steps), k-fold cross-validation was performed on an 8 channels x 41 timepoints (200 ms) feature vector. K was set to 5 and 5 repetitions were included to provide stability across different partitions of training and testing trials. Unequal trial numbers for object- vs. scene-related cues were handled by oversampling the minority class, i.e. a random sub-selection of trials from the smaller category was duplicated to match the number of trials in the larger category. Critically, this was only done during training and not during testing, avoiding any bias in cross-validation but using the whole range of data. The outcome measure was % area under the curve (AUC) of a classification Receiver Operating Characteristic (ROC), shown to be more sensitive than simple accuracy measures [S3, S4]. Chance classification would be 50%. Each participant thus provides a single cue-locked classification time course. Second-level significance was tested via FieldTrip's cluster-based permutation method (correcting for multiple comparisons across time), where initial clusters were obtained by one-tailed t-test of classification exceeding chance levels. Results show significant above-chance classification from 1.79 s to 1.99 s post cue onset ($P<.05$, corrected; red line), replicating the RSA

effects shown in Figure 4A. Note that classification is also significantly above chance when using the exact time window of significant RSA effects (1.76s-2.06s, $t(26)=2.9$, $P=.004$, one-tailed).

	T1 (%)				
	Hit	*Miss*	*CR*	*FA*	*d'*
Nap	84.04 (± 2.10)	15.96 (± 2.10)	96.37 (± 0.69)	3.63 (± 0.69)	2.89 (± 0.10)
Wake	89.74 ± (1.71)	10.26 ± (1.71)	96.42 (± 0.77)	3.58 (± 0.77)	3.15 (± 0.14)
	T2 (%)				
	Hit	*Miss*	*CR*	*FA*	*d'*
Nap	77.70 (± 2.54)	22.30 (± 2.54)	96.67 (± 0.72)	3.33 (± 0.72)	2.67 (± 0.11)
Wake	79.32 (± 3.27)	20.68 (± 3.27)	94.95 (± 1.98)	5.05 (± 1.98)	2.78 (± 0.16)
	T3 (%)				
	Hit	*Miss*	*CR*	*FA*	*d'*
Nap	79.74 (± 2.86)	20.26 (± 2.86)	96.52 (± 0.87)	3.48 (± 0.87)	2.83 (± 0.10)
Wake	80.11 (± 3.38)	19.89 (± 3.38)	94.95 (± 2.46)	5.05 (± 2.46)	2.78 (± 0.11)

Table S1: Adjective Recognition, Related to Behavioural Results The data (means ± SEM) refer to hits, misses, correct rejections (CR) and false alarms (FA) at each test phase. The sensitivity index (*d'*) [Normalized (hits/(hits + misses)) – Normalized (false alarms/(false alarms + correct rejections))] was calculated for adjective recognition memory. We adopted a log-linear approach to safeguard this analysis against errors arising from 0 and 1 values: for each participant, 0.5 was added to the total hits and total false alarms, and 1 was added to the total signal (old) trials and total noise (new) trials [S1]. Adjective recognition scores (*d'*) were applied to a 3 (Test: T1/T2/T3) X 2 (Group: Nap/Wake) mixed ANOVA. Recognition performance initially declined between T1 and T2, but then stabilised between T2 and T3 (Test main effect [Huynh-Feldt corrected]: $F(1.78,78.41)=9.00$, $P=.001$). However, these changes in performance were not modulated by the type of offline activity (Group*Session interaction [Huynh-Feldt corrected]: $F(1.78,78.41)=2.36$, $P=.11$). There was also no main effect of Group ($F(1,44)=0.53$, $P=.47$), indicating that overall adjective recognition performance was equivalent in the nap and wake groups.

All	*T1 (%)*	*T2 (%)**		*T3 (%)***	
		Not Cued	*Cued*	*Not Cued*	*Cued*
Nap	50.15 (± 2.97)	79.57 (± 2.14)	80.69 (± 2.45)	88.48 (± 1.58)	94.36 (± 0.84)
Wake	46.84 (± 2.89)	64.17 (± 4.10)	64.75 (± 3.66)	82.52 (± 3.61)	82.76 (± 3.14)

Objects	*T1 (%)*	*T2 (%)**		*T3 (%)***	
		Not Cued	*Cued*	*Not Cued*	*Cued*
Nap	48.96 (± 2.92)	80.86 (± 2.36)	80.20 (± 3.16)	90.09 (± 2.03)	96.14 (± 1.76)
Wake	47.37 (± 3.51)	67.47 (± 4.51)	68.01 (± 4.32)	84.54 (± 4.02)	88.72 (± 3.55)

Scenes	*T1 (%)*	*T2 (%)**		*T3 (%)***	
		Not Cued	*Cued*	*Not Cued*	*Cued*
Nap	51.33 (± 3.18)	78.14 (± 2.70)	80.78 (± 2.69)	87.77 (± 1.85)	92.27 (± 1.50)
Wake	46.32 (± 2.77)	61.40 (± 4.57)	61.61 (± 4.53)	79.14 (± 4.90)	76.14 (± 5.62)

Table S2: Category Recall, Related to Behavioural Results Data (means ± SEM) are shown for all items (top) and then separated for object and scenes. *Refers to the proportion (%) of T1-recalled categories that were also recalled at T2. **Refers to the proportion (%) of T2-recalled categories that were also recalled at T3. Note: 50% accuracy is not to be mistaken as chance performance given that participants had a "?" response option (see Figure 1A). We repeated the analyses reported in the main text to examine objects and scenes separately. A 2 (Type: Object/Scene) X 2 (Group: Nap/Wake) mixed ANOVA showed that object and scene recall did not differ at T1 (Type main effect: $F(1,44)=0.23$, $P=.64$), for either the nap group or the wake group (Type*Group interaction: $F(1,44)=1.54$, $P=.22$). Again, there was no main effect of Group ($F(1,44)=0.59$, $P=.45$). At T3, a main effect of Type $F(1,44)=6.88$, $P=.01$) indicated that objects were generally better recalled than scenes. However, there was no interaction between Type and any other factor(s) (all $P>.05$), suggesting that the memory effects of sleep and TMR observed in this study did not vary according to category membership. Objects were also better recalled than scenes at T2 (Type main effect: $F(1,44)=4.11$, $P=.05$). Again, there was no interaction between Type and any other factor(s) (all $P>.05$).

All	*T1 (%)*	*T2 (%)**		*T3 (%)***	
		Not Cued	*Cued*	*Not Cued*	*Cued*
Nap	83.48<br>(± 2.10)	84.93<br>(± 1.98)	84.54<br>(± 2.01)	94.06<br>(± 1.11)	96.43<br>(± 0.66)
Wake	77.35<br>(± 4.21)	68.45<br>(± 4.18)	70.74<br>(± 4.20)	89.17<br>(± 3.94)	87.52<br>(± 2.58)

Objects	*T1 (%)*	*T2 (%)**		*T3 (%)***	
		Not Cued	*Cued*	*Not Cued*	*Cued*
Nap	86.01<br>(± 2.29)	87.16<br>(± 2.41)	85.16<br>(± 2.78)	94.31<br>(± 1.64)	98.32<br>(± 0.82)
Wake	79.71<br>(± 4.32)	73.36<br>(± 4.85)	73.72<br>(± 4.89)	91.21<br>(± 3.61)	94.96<br>(± 2.39)

Scenes	*T1 (%)*	*T2 (%)**		*T3 (%)***	
		Not Cued	*Cued*	*Not Cued*	*Cued*
Nap	81.02<br>(± 2.45)	82.48<br>(± 2.61)	82.90<br>(± 2.22)	93.66<br>(± 1.43)	94.53<br>(± 1.33)
Wake	75.09<br>(± 4.44)	64.51<br>(± 5.05)	68.38<br>(± 5.15)	84.97<br>(± 5.61)	76.40<br>(± 7.22)

Table S3: Exemplar Recall, Related to Behavioural Results Data (means ± SEM) are shown for all items (top) and then separated for object and scenes. *Refers to the proportion (%) of T1-recalled items that were also recalled at T2. **Refers to the proportion (%) of T2-recalled items that were also recalled at T3. Note: the exemplar recall analysis was restricted to trials for which the category (object or scene) was correctly retrieved. At T1, more object exemplars were recalled than scene exemplars (Type (Object/Scene) main effect: F(1,44)=8.92, P=.005). There was no main effect of Group (Nap/Wake) (F(1,44)=1.99, P=.17) and no Type*Group Interaction (F(1,44)=0.01, P=.91). At T2, the nap group strongly outperformed the wake group (Group main effect: F(1,44)=18.46, P<.0001) and objects were better recalled than scenes (Type main effect: F(1,44)=7.37, P=.009). There was no main effect of TMR (F(1,44)=0.07, P=.80) and no interactions between factors (P>.05). At T3, there was again a main effect of Group (F(1,44)=7.90, P=.007) and a main effect of Type (F(1,44)=9.64, P=.003). There was also a Type*Group Interaction (F(1,44)=4.67, P=.04): objects were better recalled than scenes in the wake group but not in the nap group. While the TMR*Group interaction did not reach significance (F(1,44)=2.57, P=.12) in this analysis, planned t-tests revealed a strong trend towards superior recall for cued relative to non-cued items in the nap group (t(26)=2.01, P=.06, two-tailed), but not in the wake group (t(18)=0.48, P=.64). Taken together with our other findings, these results suggest that category level recall is most sensitive to the memory-enhancing effects of TMR. There were no other interactions between factors (P>.05).

**A**	*N1*	*N2*	*N3*	*REM*	*TST*
	14.52<br>(± 1.38)	35.78<br>(± 3.09)	14.85<br>(± 2.32)	20.70<br>(±2.84)	90.89<br>(± 2.47)

**B**	*Overall*	*F3*	*F4*	*C3*	*C4*	*P3*	*P4*	*O1*	*O2*
Slow Spindle Density	2.00<br>(± 0.10)	2.80<br>(± 0.18)	2.74<br>(± 0.17)	2.12<br>(± 0.14)	2.04<br>(± 0.14)	1.66<br>(± 0.09)	1.59<br>(± 0.10)	1.52<br>(± 0.06)	1.50<br>(± 0.06)
Fast Spindle density	3.37<br>(± 0.09)	3.10<br>(± 0.10)	3.04<br>(± 0.12)	3.70<br>(± 0.09)	3.45<br>(± 0.14)	3.92<br>(± 0.10)	3.66<br>(± 0.17)	2.99<br>(± 0.16)	3.12<br>(± 0.14)
SO *Density*	2.69<br>(± 0.08)	2.63<br>(± 0.11)	2.68<br>(± 0.10)	2.60<br>(± 0.09)	2.64<br>(± 0.08)	2.64<br>(± 0.10)	2.65<br>(± 0.08)	2.82<br>(± 0.08)	2.88<br>(± 0.07)

Table S4: Sleep EEG data, Related to EEG Results A. Time (min) spent in each stage of sleep and total sleep time (TST). **B.** Event density measures for slow spindles (10-13 Hz), fast spindles (13-16 Hz) and slow oscillations (SOs, 0.5-2 Hz) at each EEG channel. Density is calculated as the number of electrophysiological events divided by time (min) spent in non-rapid eye movement (NREM) sleep stages N2 and N3. Data are shown as means ± SEM.

Supplemental References

S1. Hautus, M.J. (1995). Corrections for extreme proportions and their biasing effects on estimated values of d'. Behavior Research Methods, Instruments & Computers 27, 46-51.

S2. Iber, C., Ancoli-Israel, S., Chesson, A., and Quan, S.F. (2007). The AASM Manual for the Scoring of Sleep and Associated Events: Rules, Terminology and Technical Specification. (Westchester (IL): American Academy of Sleep Medicine).

S3. Bradley, A.P. (1997). The Use of the Area Under the ROC Curve in the Evaluation of Machine Learning Algorithms. Pattern Recognition 30, 1145-1159.

S4. Rissman, J., Greely, H.T., and Wagner, A.D. (2010). Detecting individual memories through the neural decoding of memory states and past experience. Proceedings of the National Academy of Sciences 107, 9849-9854.
