# Supplementary material for: Memory Consolidation Is Linked to Spindle-Mediated Information Processing during Sleep
Source: Curr Biol. 2018 Mar 19;28(6):948–954.e4. doi: 10.1016/j.cub.2018.01.087 (PMC5863764; doi:10.1016/j.cub.2018.01.087)
Supplement: Document S2. Article plus Supplemental Information [file mmc2.pdf]

Report

Current Biology

Memory Consolidation Is Linked to Spindle-Mediated Information Processing during Sleep

Highlights

- We cued memory reactivation in sleep to investigate the functional role of spindles
- Memory cueing bolstered retrieval performance the following day
- Relative to control stimuli, memory cues evoked a surge in fast spindle activity
- Memory content could be reliably decoded during this spindle increase

Authors

Scott A. Cairney, Anna á Váli Guttesen, Nicole El Marj, Bernhard P. Staresina

Correspondence

b.staresina@bham.ac.uk

In Brief

Sleep spindles play a crucial role in memory consolidation, but the underlying mechanisms are not well understood. Using an auditory memory-cueing technique and EEG analysis in humans, Cairney et al. show that sleep spindles mediate the informational content of reactivated memory traces in service of offline mnemonic processing.

Cairney et al., 2018, Current Biology *28*, 948–954
March 19, 2018 © 2018 The Authors. Published by Elsevier Ltd.
https://doi.org/10.1016/j.cub.2018.01.087

CellPress

CellPress
Current Biology
Report

Memory Consolidation Is Linked to Spindle-Mediated Information Processing during Sleep

Scott A. Cairney,[1] Anna á Váli Guttesen,[1] Nicole El Marj,[2] and Bernhard P. Staresina[2,3,*]
[1]Department of Psychology, University of York, York, Y010 5DD, UK
[2]School of Psychology, University of Birmingham, Birmingham, B15 2TT, UK
[3]Lead Contact
*Correspondence: b.staresina@bham.ac.uk
https://doi.org/10.1016/j.cub.2018.01.087

SUMMARY

How are brief encounters transformed into lasting memories? Previous research has established the role of non-rapid eye movement (NREM) sleep, along with its electrophysiological signatures of slow oscillations (SOs) and spindles, for memory consolidation [1–4]. In related work, experimental manipulations have demonstrated that NREM sleep provides a window of opportunity to selectively strengthen particular memory traces via the delivery of auditory cues [5–10], a procedure known as targeted memory reactivation (TMR). It has remained unclear, however, whether TMR triggers the brain's endogenous consolidation mechanisms (linked to SOs and/or spindles) and whether those mechanisms in turn mediate effective processing of mnemonic information. We devised a novel paradigm in which associative memories (adjective-object and adjective-scene pairs) were selectively cued during a post-learning nap, successfully stabilizing next-day retention relative to non-cued memories. First, we found that, compared to novel control adjectives, memory cues evoked an increase in fast spindles. Critically, during the time window of cue-induced spindle activity, the memory category linked to the verbal cue (object or scene) could be reliably decoded, with the fidelity of this decoding predicting the behavioral consolidation benefits of TMR. These results provide correlative evidence for an information processing role of sleep spindles in service of memory consolidation.

RESULTS

We tested the prediction that TMR delivered in non-rapid eye movement (NREM) sleep bolsters memory retention by exploiting the brain's endogenous consolidation mechanisms. Of the neural oscillations unique to NREM sleep, spindles have been recently implicated in memory reactivation and spontaneous information processing [11, 12] and thus appear to be the prime candidate to mediate consolidation in a targeted manner.

As shown in Figure 1, participants (n = 46) encoded pairwise associations (adjective-object and adjective-scene pairs) before an initial test phase (T1), in which adjective-recognition judgements (old or new) were made (see Table S1). Critically, for recognized adjectives, recall of the associated image (object or scene) was assessed. Half of the correctly recalled adjective-object and adjective-scene pairs were randomly assigned to a cued condition, such that the adjectives would be replayed during the offline period (targeted memory reactivation [TMR]). The other half of the correctly recalled pairs were assigned to a non-cued condition. The to-be-cued adjectives were intermixed with a number of non-encoded control adjectives. Immediately after T1, participants either took a 90 min nap (nap group; n = 27, 19 females) or remained awake for the same period of time (wake group; n = 19 females). In the nap group, TMR took place during NREM stages N2 and N3. In the wake group, TMR coincided with a working memory task to prevent participants from directly attending to the cues [7, 9]. After the offline period, participants completed a second test (T2) before returning after an additional night of sleep to complete a final test phase (T3).

Behavior

Category recall at T1 did not differ between the nap and wake groups ($t(44) = 0.77$, $p = 0.45$; see Table S2, Figure 2A). Category retention at T2 (i.e., the proportion of T1-recalled categories that were also recalled at T2) was significantly better after sleep than wakefulness (Group main effect: $F(1,44) = 17.10$; $p < .0001$) but unaffected by cueing (TMR main effect: $F(1,44) = 0.19$, $p = 0.66$; TMR*Group interaction: $F(1,44) = 0.02$, $p = 0.89$) (Figure 2C). However, category retention at T3 (i.e., the proportion of T2-recalled categories that were also recalled at T3) revealed both a memory benefit of sleep (Group main effect: $F(1,44) = 9.34$; $p = 0.004$) and a recall advantage from cueing (TMR main effect: $F(1,44) = 4.65$, $p = 0.04$; TMR*Group interaction: $F(1,44) = 3.94$, $p = 0.05$). The memory-enhancing effects of TMR were driven by the nap group, who exhibited superior retention of cued relative to non-cued categories ($t(26) = 3.83$, $p = 0.001$), whereas no such difference was observed in the wake group ($t(18) = 0.09$, $p = 0.93$). Taken together, these results suggest that TMR in post-learning sleep augmented overnight consolidation processes, improving retention the following day.

 © 2018 The Authors. Published by Elsevier Ltd.
This is an open access article under the CC BY license (http://creativecommons.org/licenses/by/4.0/).

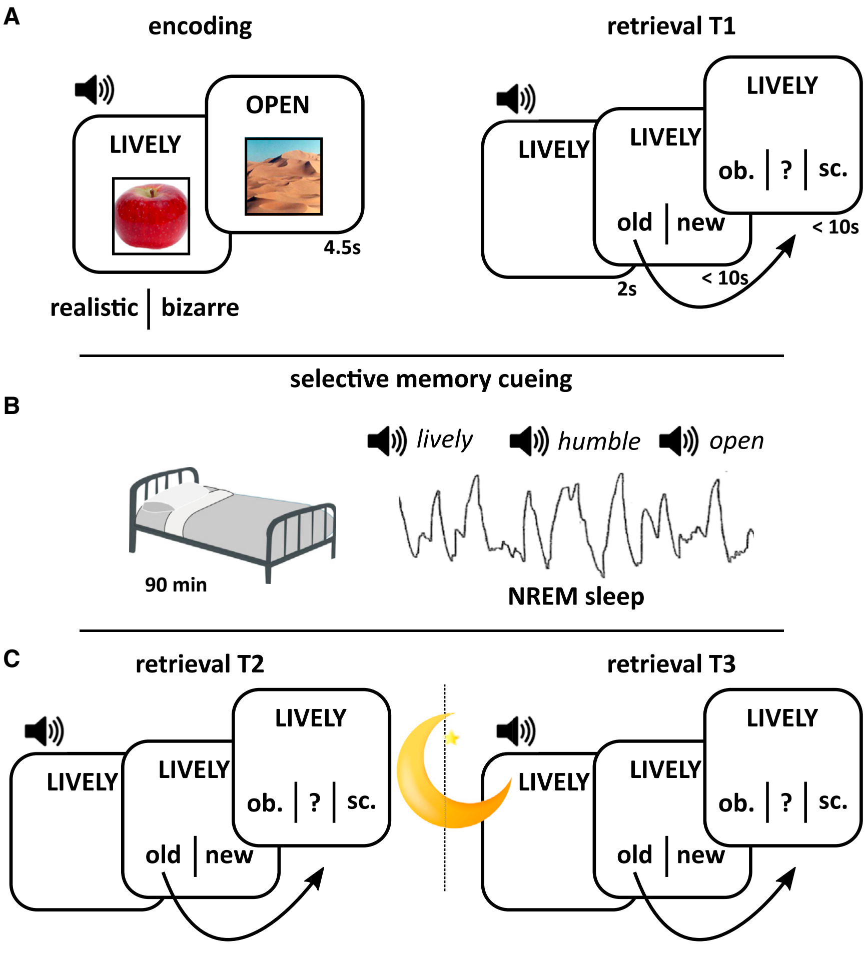

Figure 1. Experimental Paradigm

(A) During encoding, participants were presented with 50 adjective-object and 50 adjective-scene combinations (randomly intermixed) and indicated whether the combinations elicited a realistic or bizarre mental image. Prior to encoding, participants performed a familiarization phase for both the adjectives and the images (see STAR Methods). Approximately 5 min after encoding, participants performed the first retrieval session (T1), in which all previously seen (old) adjectives were intermixed with 50 previously unseen (new) adjectives and participants indicated whether they thought the adjective was old or new. In the case of an "old" response, participants were asked whether they also remembered the associated image category (object or scene) or whether they did not remember the associated category ("?" response option). If they indicated "object" or "scene," another screen appeared (not shown) in which participants could type in a description of the image if they remembered it or simply type in a "?" if they did not. Adjectives were presented visually and acoustically throughout.

(B) In the nap group, participants were given the opportunity to sleep for 90 min (monitored with polysomnography). Once they entered late NREM sleep (stages N2 and N3), (1) half of the adjectives for which the object category was remembered at T1, (2) half of the adjectives for which the scene category was remembered at T1, and (3) a matched number of novel adjectives (controls) were continuously played via external speakers (targeted memory reactivation [TMR]). In the wake group, participants started with 30 min of playing the online game Bubble Shooter, followed by 30 min of performing a 1-back working memory task during which TMR was applied, followed again by 30 min of playing Bubble Shooter.

(C) After the offline period (T2), participants performed the same test as in T1 but with a new set of 50 lure adjectives. Finally, after a night of sleep, participants returned the next morning (T3) for another retrieval session, again with 50 new lure adjectives. For detailed description of behavioral results, see Tables S1–S3.

In the nap group, the behavioral benefit of TMR on T3 retention, quantified as: [proportion of cued T2-recalled categories also recalled at T3 minus proportion of non-cued T2-recalled categories also recalled at T3] was not correlated with the total number of memory cues presented (objects + scenes) in sleep (Spearman's rho = 0.02, p = 0.93; see STAR Methods). Exemplar recall data is available in Table S3.

At the end of the experiment, participants were re-presented with all of the adjectives from the encoding phase and, for each, asked to indicate whether or not it was replayed in the offline period. The discrimination task analysis was restricted to items that were correctly recalled at T1 (and thus assigned to the cued and non-cued conditions). Cued stimuli that were and were not correctly identified as such were marked as hits and misses, respectively. Non-cued stimuli that were and were not correctly identified as such were marked as correct rejections or false alarms, respectively. A discrimination index was then calculated for each participant by subtracting the proportion of non-cued trials marked as false alarms from the proportion of cued trials marked as hits. The discrimination index was not significantly different from zero in either the nap group ($t(26)$ = 0.07, p = 0.95) or the wake group ($t(18)$ = 1.67, p = 0.11), implying that participants could not reliably identify the cue stimuli. It should be noted that none of the nap group participants professed any knowledge of adjective replay.

EEG

As a first step, we explored the event-related potentials (ERPs) evoked by auditory cues for previously presented (old) adjectives in the nap group (see Table S4 for descriptive sleep electroencephalogram [EEG] data). As shown in Figure 2B, auditory cues were followed by a pronounced ERP response resembling a slow oscillation (SO)/k-complex, with comparable patterns for old object cues, old scene cues, and new control stimuli (Figure S1). Note also that the relatively small ERP amplitude results from the temporal jitter of evoked responses relative to the cue onset and that these responses surpass standard amplitude criteria for SOs/k-complexes when aligned to their respective event centers (Figure S2). Consistent with previous findings [13], time-frequency representation (TFR) results showed that these cue-induced SOs harbored a theta/slow spindle burst in the SO trough (henceforth referred to as SO down-state), which was followed by a fast spindle burst in the ensuing SO peak (henceforth referred to as SO up-state).

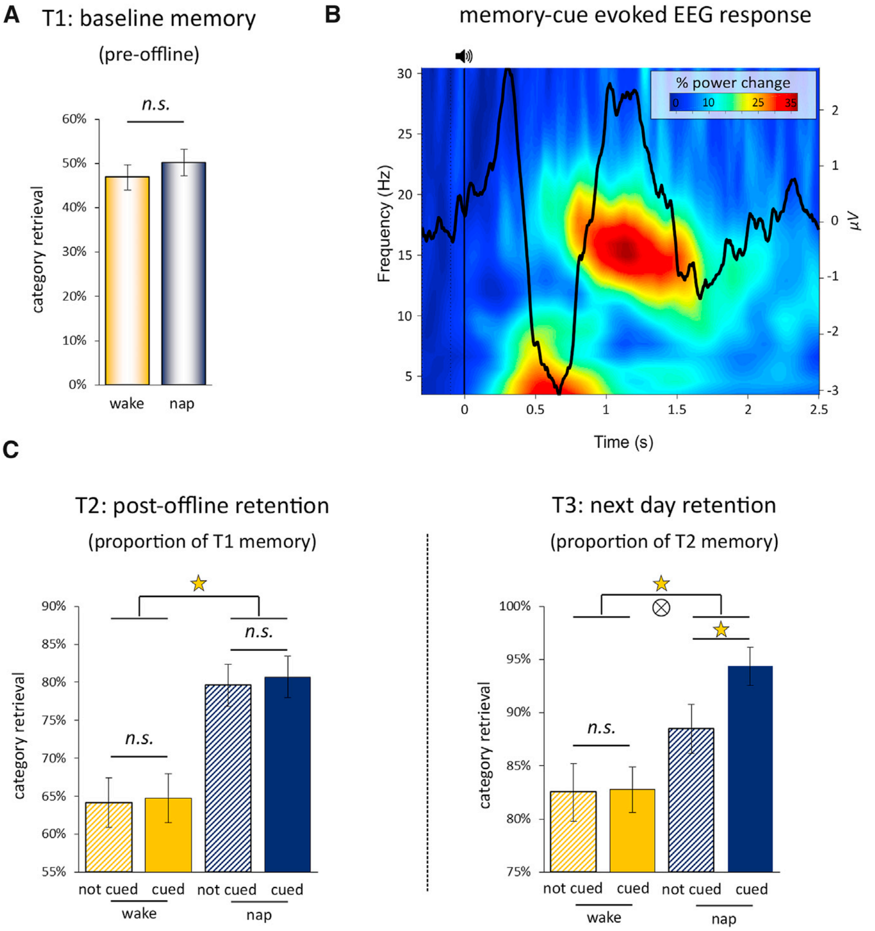

Figure 2. Behavior and Evoked Responses

(A) Behavioral results at T1 (pre-offline period). Bar graphs show mean (±SEM) accuracy for adjective-category retrieval for the nap group (blue) and the wake group (orange). Note that 50% accuracy is not to be mistaken as chance performance given that participants had a "?" response option (see Figure 1A).

(B) Event-related potential (ERP) and time-frequency representation (TFR) evoked by the onset of memory cues. The figure depicts the unthresholded TFR along with the grand average ERP (both collapsed across all channels and then averaged across participants), revealing a strong increase of theta/slow spindle power in the evoked SO down-state followed by an increase in fast spindle power in the ensuing SO up-state. ERP topographies for object, scene, and control stimuli are shown in Figure S1.

(C) At T2 and T3, behavioral results are further separated into cued trials (solid fill) and not cued trials (hatched fill), and retrieval accuracy is expressed as proportions retained from the most recent memory assessment (see also Table S2). Stars denote significant effects, ⊗ denotes an interaction effect.

To more directly isolate the mechanisms engaged in the processing of mnemonic cues, we next asked whether evoked oscillatory responses might be able to distinguish between old cues and novel control adjectives. As shown in Figures 3A and 3B, the direct contrast revealed that old cues elicited a significant increase in oscillatory power in the fast spindle band (13–16 Hz) from ~1.7 to 2.3 s after cue onset ($p < 0.05$, corrected for multiple comparisons across channels, time, and frequency). Topographical representation of the significant spindle band increase revealed that the effect stemmed from left-hemisphere electrodes, with a maximum at centroparietal sites C3/P3 (Figure 3C). The increase in fast spindle power for old cues versus control stimuli (1.7–2.3 s; Figure 3B) was even more pronounced when restricting old cues to those that yielded successful memory during both T2 and T3 testing ($t(26) = 2.10$, $p = 0.007$ as opposed to $t(26) = 2.09$, $p = 0.014$ when including all trials).

To fully characterize the observed spindle power increase for old (versus new) cues, we algorithmically detected discrete spindle events (see STAR Methods) from 1.5 to 2.5 s post-cue onset (set to encapsulate the window of increased spindle power in our TFR analysis). Indeed, old relative to new cues elicited more fast spindles across left-hemisphere electrodes ($t(26) = 3.33$, $p = 0.003$, Figure S3A), corroborating that the spindle band power increase (Figure 3A) is due to a relative surge in discrete spindle events. Figure 3D displays the resulting grand average spindle across participants, aligned to the maximum of the detected spindle events. As can be appreciated by the above-zero spindle center, these spindles appeared to be modulated by the up-states of concomitant SOs. To statistically quantify this observation, we derived the preferred phase of spindle-SO coupling for the detected events (see STAR Methods). Indeed, as shown in Figure 3D, the preferred phase of SO-spindle modulation clustered significantly around the SO up-state (0°) across participants (Rayleigh test: $z = 8.7$, $p < .001$; V test versus 0: $v = 13.9$, $p < .001$). In sum, these results show that old memory cues relative to new control adjectives elicit an increase in fast spindle events, which are localized to left-hemisphere sites. Spindles were further found to be modulated by the SO up-state, but note that direct comparison of SO-spindle coupling for old cues versus control adjectives was impeded by the low numbers of spindles elicited by control adjectives.

If the surge in fast spindles for old cues was indeed indicative of cue-induced information processing, we reasoned that we should be able to decode from evoked EEG responses the categorical features of the images paired with the adjectives during learning (object versus scene; Figure 1). We used a representational similarity analysis (RSA) approach to tackle this question [14]. First, we derived a feature vector of 8 channels × 41 time points (spanning 200 ms at our sampling rate of 200 Hz) centered at each sample from −0.2 to 2.5 s relative to cue onset. Next, using Spearman correlations, we quantified both the within-category similarity (how similar is the EEG pattern of a given object-related adjective to the EEG pattern of all other object-related adjectives, and how similar is the pattern of a given scene-related adjective to the pattern of all other scene-related

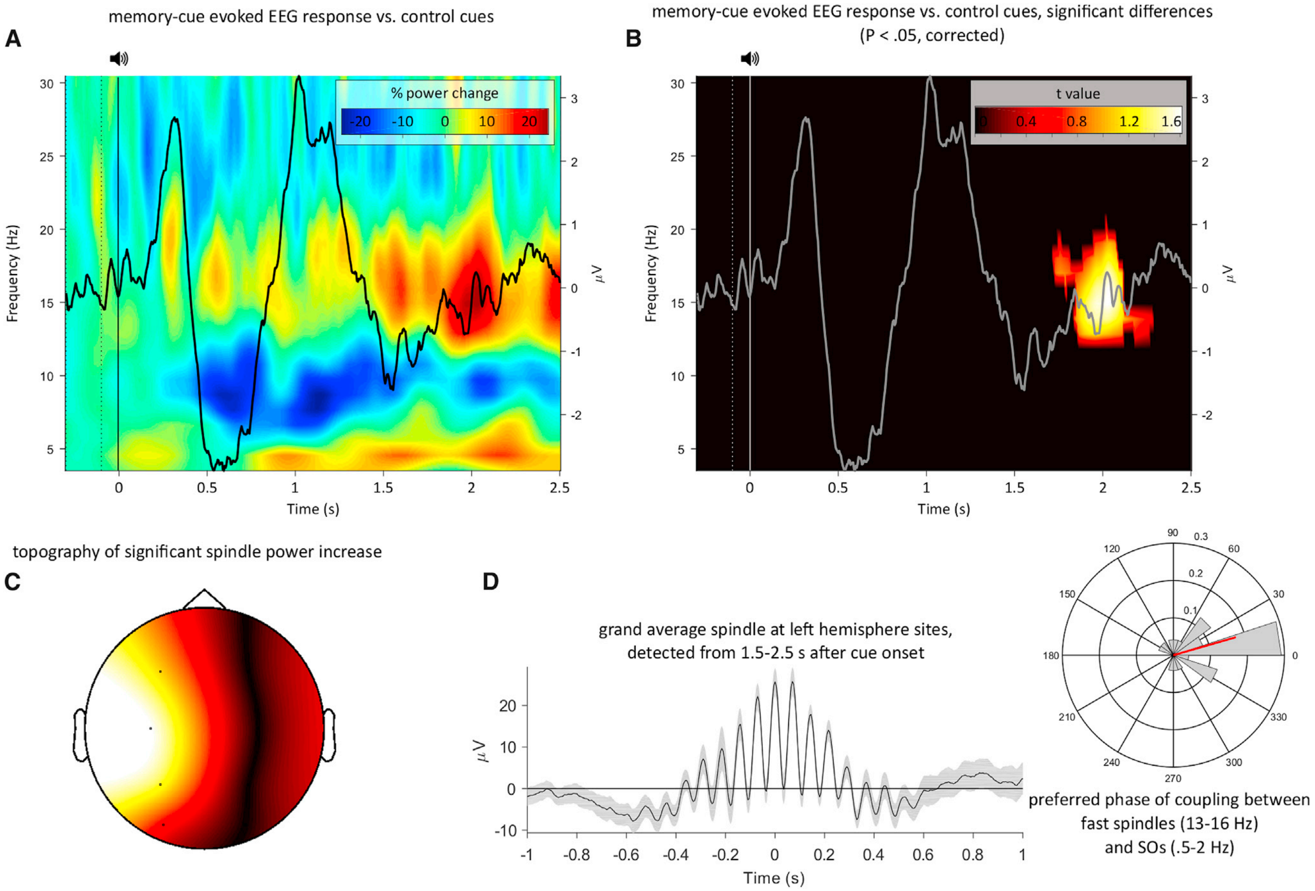

Figure 3. Time-Frequency Representation

(A) Time-frequency representation (TFR) difference map of responses elicited by old memory cues versus new control adjectives, with the corresponding ERP for old cues superimposed.

(B) Same as (A) but after statistical thresholding ($p < 0.05$, corrected). Note the significant increase in fast spindle power (13–16 Hz) from ~1.7 to 2.3 s post cue onset.

(C) Topography of the resulting cluster, revealing left-hemisphere specificity of the effect.

(D) Left: Grand average (±SEM) of discrete spindle events detected from 1.5 to 2.5 s after onset of old memory cues at left-hemisphere sites. Right: Histogram of participants' corresponding SO-spindle modulation phases (mean direction = 15°, shown in red). Direct comparison of discrete spindles for old memory cues versus new control stimuli is shown in Figure S3.

adjectives) and the between-category similarity (how similar is the pattern of a given object-related adjective to the pattern of all scene-related adjectives and vice versa) at each time point. Converging evidence was sought via a standard decoding approach (linear discriminant analysis [LDA]; see Figure S4). However, the advantage of our RSA approach is that it provides, via the between-category similarity, a single measure that captures the level of pattern distinctiveness of objects versus scenes (i.e., the smaller the between-category similarity, the greater the category distinctiveness of object versus scene information).

Category-specific information processing would be expressed as an increase in within-category similarity (collapsed across object- and scene-related adjectives) compared to between-category similarity. As shown in Figure 4A, within-category similarity tended to exceed between-category similarity throughout the post-cue period. Importantly, however, the strongest effect that also reached statistical significance ($p < .05$, corrected for multiple comparisons across time) was observed from 1.76 to 2.06 s after cue onset, which is closely overlapped with the observed window of increased fast spindle power for old relative to new cues (1.7–2.3 s; Figure 3B). It deserves explicit mention that the EEG features used for this analysis was not spindle power per se but the raw EEG trace across electrodes (see STAR Methods). In fact, the diagnostic information was largely carried by lower-frequency topographies; i.e., the result pattern in Figure 4A held when low pass filtering the EEG below 4 Hz but diminished when high pass filtering the EEG above 4 Hz. This could be due to spindles effectively inducing event-related (lower frequency) EEG responses at target sites and/or to the fact that higher-frequency EEG components are too irregular to allow reliable time-by-time decoding across trials. In any case, to investigate the relationship between fast spindles and category-specific information processing beyond temporal co-occurrence, we next assessed the correlation between (1) relative spindle counts for old cues versus control stimuli from 1.5 to 2.5 s post-cue and (2) the level of category distinctiveness

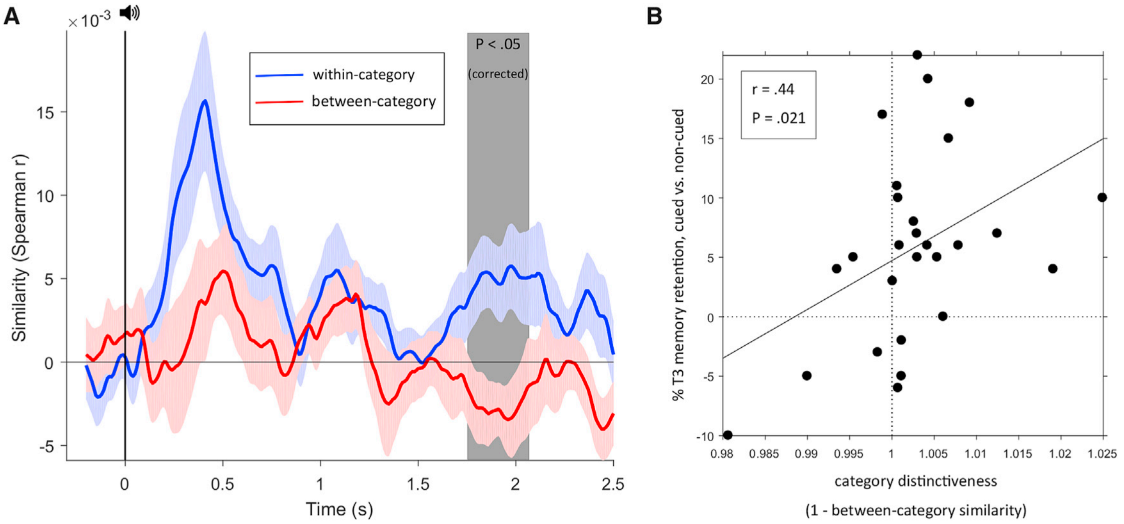

Figure 4. Information Processing Evoked by Memory Cues

(A) Time courses (mean ± SEM) of within- and between-category similarities in response to old memory cues. Shaded area from 1.76 to 2.06 s highlights a significant increase ($p < .05$, corrected for multiple comparisons across time, see also Figure S4).

(B) Spearman correlation of category distinctiveness (1 minus between-category similarity from 1.76 to 2.06 s) and the behavioral benefit of cueing on overnight consolidation across participants.

for objects versus scenes (1 minus between-category similarity) in the same time window. Intriguingly, we observed a significant positive correlation between the two variables (Spearman's rho = 0.47, p = 0.014; Figure S3B), supporting the notion that spindles are linked to processing of the informational content of reactivated memory traces.

Lastly, we asked whether spindle-mediated information processing bears any relevance for behavioral manifestations of consolidation. To this end, we assessed the correlation between (1) the behavioral benefit of cueing on T3 retention (proportion of cued T2-recalled categories also recalled at T3 minus proportion of non-cued T2-recalled categories also recalled at T3) and (2) category distinctiveness of object versus scene information (1 minus between-category similarity, averaged across the 1.76–2.06 s effect window). As shown in Figure 4B, the results revealed a significant positive relationship between the two variables (Spearman's rho = 0.41, p = 0.03).

DISCUSSION

Our study reveals that spindle-mediated information processing is a central mechanism for offline consolidation and that TMR may exploit this mechanism to selectively strengthen particular memory traces. Specifically, we found that memory cues delivered in NREM sleep prompted a transient increase in fast spindles that was coupled to depolarising SO up-states (Figure 3). During this surge in spindle activity, the categorical features of cued representations could be reliably decoded, with the level of category distinctiveness predicting the behavioral impact of TMR on consolidation (Figure 4).

What is the functional significance of fast spindles for memory consolidation? Simultaneous EEG-fMRI recordings have shown that reactivation of learning networks is linked to spindle parameters during subsequent NREM sleep [11]. Moreover, olfactory memory cueing has been shown to evoke sleep spindles in task-relevant brain regions [15]. Although ceiling memory performance in these two studies precluded a direct link to consolidation, their findings suggest that a TMR-induced increase in fast spindle activity may reflect mnemonic processing in relevant hippocampal-neocortical networks. This view is substantiated by the left-hemispheric specificity of the cue-evoked spindle increase observed in our study (Figure 3C), which may reflect the verbal properties of the auditory cues.

More critically, during this transient, cue-induced fast spindle increase, we were able to reliably decode the categorical features (i.e., object versus scene) of the mnemonic association linked to the verbal cue (Figures 4A and S4), with the fidelity of category distinctiveness predicting the cueing benefit on next-day memory retention across participants (Figure 4B). One intriguing possibility is that spindles effectively gate activation toward category-specific cortical modules, leading to discriminable distributions of the spatiotemporal EEG patterns. Consistent with this notion, recent work applying EEG classifiers to continuous overnight sleep recordings has shown that spectral power in the spindle range contributes to the ability to decode previously learned materials [12]. It would thus be interesting for future studies to directly compare the neural correlates of cue-evoked versus spontaneous memory reactivation. Also, both studies provide correlational rather than causal evidence for spindle-mediated information processing, and electrophysiological control over various spindle parameters would strongly corroborate the relationship between spindles and memory consolidation.

Mechanistically, modeling and empirical data suggest that spindle oscillations induce a massive Ca^{2+} influx into dendrites of pyramidal neurons, opening a molecular "gate" for synaptic plasticity and, consequently, permanent network changes [16–18]. Finely tuned windows of spindle activity, triggered by

TMR, may therefore prime or "tag" relevant synapses for plastic changes during subsequent periods of sleep. Owing to the highly robust effects of sleep (versus wake) on retention in the current paradigm (Figure 2C), however, the comparatively subtle mnemonic influences of TMR may have failed to emerge immediately after sleep. This may explain why the behavioral benefits of TMR observed in this study did not emerge until the following day, once the cued (tagged) representations had undergone additional overnight processing. It is important to note that the time between TMR and the overnight sleep interval included an intervening test phase (T2; Figure 1C). Given recent views on the potential mechanistic overlap between online reactivation and offline consolidation [19], it is also possible that T2-retrieval modulated interactions between TMR and subsequent overnight memory processing. In other words, the reactivation of memories at T2 might have contributed to the observable benefit of cueing at T3. How the memory effects of TMR are influenced by memory reprocessing during online and offline periods will be a fruitful avenue for future research.

Owing to the limited spatial resolution of scalp EEG monitoring, the putative role of hippocampally generated ripples (>80 Hz oscillations) in our paradigm remains open. This is an important consideration as neuronal reactivations are mostly observed in conjunction with ripple events [20–22], which are temporally nested within the oscillatory troughs of spindles [23–25]. The *Active Systems* framework postulates that these synchronized spindle-ripple interactions enable spindle oscillations to shuttle reactivated hippocampal representations to distributed neocortical sites during excitable SO up-states [26–29]. Unifying our experimental paradigm with methods for detecting hippocampal ripples in humans (e.g., intracranial EEG) would thus provide exceptional insights into mnemonic processing in the sleeping brain. Notably, spindle-ripple interactions may reveal even greater detail on the informational content of decoded associations than spindle oscillations alone.

In sum, our findings suggest that experimental memory cueing generates finely tuned windows of spindle-mediated information processing, which underpins the selective strengthening of cued representations. These findings not only offer mechanistic insights into the mnemonic impacts of TMR but also provide unique and highly controlled experimental evidence for the critical role of spindles in offline memory consolidation.

STAR★METHODS

Detailed methods are provided in the online version of this paper and include the following:

- KEY RESOURCES TABLE
- CONTACT FOR REAGENT AND RESOURCE SHARING
- EXPERIMENTAL MODEL AND SUBJECT DETAILS
- METHOD DETAILS
  - Stimuli
  - Procedure
  - Equipment
- QUANTIFICATION AND STATISTICAL ANALYSIS
  - Data Analysis
- DATA AND SOFTWARE AVAILABILITY

SUPPLEMENTAL INFORMATION

Supplemental Information includes four figures and four tables and can be found with this article online at https://doi.org/10.1016/j.cub.2018.01.087.

ACKNOWLEDGMENTS

This research was supported by a Wellcome Trust Institutional Strategic Support Fund (105624) through a University of York Centre for Chronic Diseases and Disorders (C2D2) Fellowship and a Medical Research Council (MRC) Career Development Award (MR/P020208/1) to S.A.C. and a Wellcome Trust/Royal Society Sir Henry Dale Fellowship to B.P.S. (107672/Z/15/Z). The authors are grateful to Aidan Horner and Gareth Gaskell for fruitful discussions of the data.

AUTHOR CONTRIBUTIONS

S.A.C. and B.P.S. designed the study. A.á.V.G. and N.E.M. performed the experiments. S.A.C. and B.P.S. analyzed the data and wrote the manuscript.

DECLARATION OF INTERESTS

The authors declare no competing interests.

Received: November 10, 2017
Revised: January 10, 2018
Accepted: January 31, 2018
Published: March 8, 2018

REFERENCES

1. Marshall, L., Helgadóttir, H., Mölle, M., and Born, J. (2006). Boosting slow oscillations during sleep potentiates memory. Nature *444*, 610–613.
2. Mednick, S.C., McDevitt, E.A., Walsh, J.K., Wamsley, E., Paulus, M., Kanady, J.C., and Drummond, S.P.A. (2013). The critical role of sleep spindles in hippocampal-dependent memory: a pharmacology study. J. Neurosci. *33*, 4494–4504.
3. Ngo, H.-V.V., Martinetz, T., Born, J., and Mölle, M. (2013). Auditory closed-loop stimulation of the sleep slow oscillation enhances memory. Neuron *78*, 545–553.
4. Ngo, H.-V.V., Miedema, A., Faude, I., Martinetz, T., Mölle, M., and Born, J. (2015). Driving sleep slow oscillations by auditory closed-loop stimulation-a self-limiting process. J. Neurosci. *35*, 6630–6638.
5. Cairney, S.A., Sobczak, J.M., Lindsay, S., and Gaskell, M.G. (2017). Mechanisms of memory retrieval in slow-wave sleep. Sleep *40*, https://doi.org/10.1093/sleep/zsx114.
6. Fuentemilla, L., Miró, J., Ripollés, P., Vilà-Balló, A., Juncadella, M., Castañer, S., Salord, N., Monasterio, C., Falip, M., and Rodríguez-Fornells, A. (2013). Hippocampus-dependent strengthening of targeted memories via reactivation during sleep in humans. Curr. Biol. *23*, 1769–1775.
7. Rudoy, J.D., Voss, J.L., Westerberg, C.E., and Paller, K.A. (2009). Strengthening individual memories by reactivating them during sleep. Science *326*, 1079.
8. Oudiette, D., Antony, J.W., Creery, J.D., and Paller, K.A. (2013). The role of memory reactivation during wakefulness and sleep in determining which memories endure. J. Neurosci. *33*, 6672–6678.
9. Schreiner, T., and Rasch, B. (2015). Boosting vocabulary learning by verbal cueing during sleep. Cereb. Cortex *25*, 4169–4179.
10. Cousins, J.N., El-Deredy, W., Parkes, L.M., Hennies, N., and Lewis, P.A. (2016). Cued reactivation of motor learning during sleep leads to overnight changes in functional brain activity and connectivity. PLoS Biol. *14*, e1002451.
11. Bergmann, T.O., Mölle, M., Diedrichs, J., Born, J., and Siebner, H.R. (2012). Sleep spindle-related reactivation of category-specific cortical regions after learning face-scene associations. Neuroimage *59*, 2733–2742.

12. Schönauer, M., Alizadeh, S., Jamalabadi, H., Abraham, A., Pawlizki, A., and Gais, S. (2017). Decoding material-specific memory reprocessing during sleep in humans. Nat. Comm. *8*, https://doi.org/10.1038/ncomms15404.
13. Schreiner, T., Lehmann, M., and Rasch, B. (2015). Auditory feedback blocks memory benefits of cueing during sleep. Nat. Commun. *6*, 8729.
14. Kriegeskorte, N., Mur, M., and Bandettini, P. (2008). Representational similarity analysis - connecting the branches of systems neuroscience. Front. Syst. Neurosci. *2*, https://doi.org/10.3389/neuro.06.004.2008.
15. Cox, R., Hofman, W.F., de Boer, M., and Talamini, L.M. (2014). Local sleep spindle modulations in relation to specific memory cues. Neuroimage *99*, 103–110.
16. Rosanova, M., and Ulrich, D. (2005). Pattern-specific associative long-term potentiation induced by a sleep spindle-related spike train. J. Neurosci. *25*, 9398–9405.
17. Sejnowski, T.J., and Destexhe, A. (2000). Why do we sleep? Brain Res. *886*, 208–223.
18. Timofeev, I., Grenier, F., Bazhenov, M., Houweling, A.R., Sejnowski, T.J., and Steriade, M. (2002). Short- and medium-term plasticity associated with augmenting responses in cortical slabs and spindles in intact cortex of cats in vivo. J. Physiol. *542*, 583–598.
19. Antony, J.W., Ferreira, C.S., Norman, K.A., and Wimber, M. (2017). Retrieval as a fast route to memory consolidation. Trends Cogn. Sci. *21*, 573–576.
20. Buzsáki, G. (2015). Hippocampal sharp wave-ripple: A cognitive biomarker for episodic memory and planning. Hippocampus *25*, 1073–1188.
21. Kudrimoti, H.S., Barnes, C.A., and McNaughton, B.L. (1999). Reactivation of hippocampal cell assemblies: effects of behavioral state, experience, and EEG dynamics. J. Neurosci. *19*, 4090–4101.
22. Nakashiba, T., Buhl, D.L., McHugh, T.J., and Tonegawa, S. (2009). Hippocampal CA3 output is crucial for ripple-associated reactivation and consolidation of memory. Neuron *62*, 781–787.
23. Clemens, Z., Mölle, M., Erőss, L., Barsi, P., Halász, P., and Born, J. (2007). Temporal coupling of parahippocampal ripples, sleep spindles and slow oscillations in humans. Brain *130*, 2868–2878.
24. Clemens, Z., Mölle, M., Erőss, L., Jakus, R., Rásonyi, G., Halász, P., and Born, J. (2011). Fine-tuned coupling between human parahippocampal ripples and sleep spindles. Eur. J. Neurosci. *33*, 511–520.
25. Staresina, B.P., Bergmann, T.O., Bonnefond, M., van der Meij, R., Jensen, O., Deuker, L., Elger, C.E., Axmacher, N., and Fell, J. (2015). Hierarchical nesting of slow oscillations, spindles and ripples in the human hippocampus during sleep. Nat. Neurosci. *18*, 1679–1686.
26. Born, J., Rasch, B., and Gais, S. (2006). Sleep to remember. Neuroscientist *12*, 410–424.
27. Born, J., and Wilhelm, I. (2012). System consolidation of memory during sleep. Psychol. Res. *76*, 192–203.
28. Diekelmann, S., and Born, J. (2010). The memory function of sleep. Nat. Rev. Neurosci. *11*, 114–126.
29. Rasch, B., and Born, J. (2013). About sleep's role in memory. Physiol. Rev. *93*, 681–766.
30. Brainard, D.H. (1997). The psychophysics toolbox. Spat. Vis. *10*, 433–436.
31. Oostenveld, R., Fries, P., Maris, E., and Schoffelen, J.M. (2011). FieldTrip: Open source software for advanced analysis of MEG, EEG, and invasive electrophysiological data. Comput. Intell. Neurosci. *2011*, 156869.
32. Berens, P. (2009). CircStat: A MATLAB Toolbox for Circular Statistics. J. Stat. Software *31*, https://doi.org/10.18637/jss.v031.i10.
33. Staresina, B.P., Duncan, K.D., and Davachi, L. (2011). Perirhinal and parahippocampal cortices differentially contribute to later recollection of object- and scene-related event details. J. Neurosci. *31*, 8739–8747.
34. Konkle, T., Brady, T.F., Alvarez, G.A., and Oliva, A. (2010). Scene memory is more detailed than you think: the role of categories in visual long-term memory. Psychol. Sci. *21*, 1551–1556.
35. Iber, C., Ancoli-Israel, S., Chesson, A., and Quan, S.F. (2007). The AASM Manual for the Scoring of Sleep and Associated Events: Rules, Terminology and Technical Specification (Westchester, IL: American Academy of Sleep Medicine).
36. Grootswagers, T., Wardle, S.G., and Carlson, T.A. (2017). Decoding dynamic brain patterns from evoked responses: a tutorial on multivariate pattern analysis applied to time series neuroimaging data. J. Cogn. Neurosci. *29*, 677–697.
37. Cox, R., Schapiro, A.C., Manoach, D.S., and Stickgold, R. (2017). Individual differences in frequency and topography of slow and fast sleep spindles. Front. Hum. Neurosci. *11*, 433.
38. Cohen, M.X. (2008). Assessing transient cross-frequency coupling in EEG data. J. Neurosci. Methods *168*, 494–499.

STAR★METHODS

KEY RESOURCES TABLE

REAGENT or RESOURCE	SOURCE	IDENTIFIER
Software and Algorithms		
MATLAB 2015a	Mathworks	https://uk.mathworks.com/
Psychtoolbox 3.0.13	[30]	N/A
Embla RemLogic 3.0	Natus Neurology	www.natus.com
SPSS Statistics 24	IBM	https://www.ibm.com/analytics/us/en/technology/spss/
Fieldtrip Toolbox v.06/02/2017	[31]	http://www.fieldtriptoolbox.org/
CircStat Toolbox v.1	[32]	https://www.jstatsoft.org/article/view/v031i10

CONTACT FOR REAGENT AND RESOURCE SHARING

Further information and requests for resources and reagents should be directed to and will be fulfilled by the Lead Contact, Bernhard Staresina (b.staresina@bham.ac.uk).

EXPERIMENTAL MODEL AND SUBJECT DETAILS

A total of 83 participants took part in this study. However, 15 participants were excluded because they did not meet the performance criterion in the pre-sleep test (T1). One further participant withdrew having not understood the necessary time commitments of the study. Of those participants remaining who took part in the nap version of the experiment, a further 21 were excluded for the following reasons: insufficient sleep such that at least one full round of targeted memory reactivation (TMR) could not be attained (9), exhibiting an arousal or awakening during TMR and not returning to non-rapid eye movement sleep stage N2/N3 (10) and computer malfunction (2). The analyses reported in this paper were thus carried out on 46 participants, who were assigned to a nap group (n = 27, 19 female, mean ± SD age, 19.70 ± 1.51 years) or a wake group (n = 19 females, mean ± SD age, 19.26 ± 1.15 years). Note that all results reported in the main text were reliable when restricting the nap group to female participants only (n = 19). Pre-study screening questionnaires indicated that participants had no history of sleep, psychiatric or neurological disorders and were not using any psychologically active medications. Participants were informed that they were taking part in a memory study, but were unaware that targeted memory reactivation (TMR) would take place. Also, no explicit mention of the T3 memory test was provided once the first session was completed. Written informed consent was obtained from all participants in line with the Research Ethics Committees of the Department of Psychology, University of York and the School of Psychology, University of Birmingham.

METHOD DETAILS

Stimuli

Adjectives

250 adjectives were randomly selected from a longer list of 355 [33] for each participant. Mean (±SD) adjective length was 6.85 ± 1.84 characters and number of syllables ranged from 1-4. All adjectives were recorded in a female voice. Mean (±SD) duration of all included adjectives was 704 ± 146 ms.

Objects and Scenes

100 images (50 objects and 50 scenes) were randomly selected from a set of 200 [34] for each participant. Objects were images of everyday items, animals or food presented on a plain white background (e.g., an apple). Scenes were images of nameable landscapes or places (e.g., a bowling alley). Care was taken to ensure that scenes contained sufficient background detail to be easily distinguishable from objects. The distinction between objects and scenes was clearly explained to participants.

Procedure

Participants completed a short practice version (10 trials) of each experimental task to ensure that they fully understood the instructions. All responses were made via keyboard press on a PC. Experimental stimuli were always presented in random order.

Familiarisation

A familiarisation phase at the beginning of the experiment was designed to facilitate learning of the adjective-image pairs in the main encoding session. First, participants completed an adjective familiarisation task. On each trial, one of 100 adjectives (e.g., ‘‘exotic’’) was presented acoustically and displayed for 2.5 s on the computer screen. Participants indicated whether they considered the adjective to be emotionally positive or negative. Each trial was separated by an inter-stimulus interval (ISI) with a fixation cross for

1.5 s (±100 ms random jitter). Next, participants completed an object/scene categorisation task. On each trial, one of 50 objects (e.g., butterfly) or 50 scenes (e.g., golf course) was displayed for 2.5 s. Participants indicated whether they considered the image to be an object or a scene (ISI = 1.5 s ± 100 ms).

Encoding

Participants learned randomized pairwise associations between the adjectives and images presented in the familiarisation phase (100 adjective-image pairs). On each trial, a randomly selected adjective (e.g., "exotic") was presented acoustically and displayed above an object or scene (e.g., object: butterfly) for 4.5 s. To facilitate learning, participants were instructed to form a vivid mental image or story that closely linked the adjective and the object/scene, and then indicate whether the image they had formed was realistic or bizarre (ISI = 1.5 s ± 100 ms). For example, the mental image corresponding to the adjective "exotic" and the object butterfly would presumably be realistic as butterflies can be exotic creatures. Participants were informed that their memory for adjective-image pairs would be tested immediately afterward.

Immediate Test (T1)

T1 included the 100 adjectives from encoding intermixed with 50 new adjectives that participants had not seen before (foils). On each trial, an adjective (e.g., "exotic") was presented acoustically and visually displayed for 2 s. Afterward, participants were asked to indicate whether the adjective was 'old' (i.e., they recognized it from the encoding phase) or 'new' (i.e., it was not seen at encoding) within 10 s. When participants provided a "new" response, they immediately moved on to the next trial (ISI = 1.5 s ± 100 ms). When an "old" response was provided, participants were required to indicate whether the associated image was an object or a scene, or whether they had forgotten the target image category. In order to ensure that object or scene responses were not mere guesses, participants also provided a typed description of the image they had remembered. Across all T1 trials where the category was correctly recalled, participants were able to correctly describe the image on the majority of occasions (mean ± SD: 80.95 ± 14.59%), demonstrating that the category responses reflected veridical memory.

TMR Set Up

Of the adjective-image pairs that were correctly recalled at T1 (i.e., when the adjective was correctly recognized and the associated image category correctly recalled), half were randomly allocated to the cued condition (i.e., for TMR), whereas the other half were assigned to the non-cued condition (object and scene pairs were equally distributed between the two conditions). This ensured that baseline category recall performance was balanced between the cued and non-cued memories. For example, if a participant correctly recalled 40 pairs at T1, then 20 of these would be assigned to the cued condition and the other 20 assigned to the non-cued condition. On occasions where there were an odd number of correctly recalled pairs, the spare item was randomly allocated to the cued or non-cued condition. To ensure that a sufficient number of stimuli were available for TMR in sleep, participants were required to correctly recall at least 14 objects and 14 scenes at T1. Participants that did not meet this criterion were excluded (n = 15). The adjectives from pairs assigned to the cued condition were replayed during the TMR phase. Importantly, an additional set of control adjectives that participants had not encountered at either encoding or T1 were randomly intermixed with the TMR stimuli. The number of control adjectives was equal to half the number of stimuli in the cued condition. For example, if there were 40 adjectives associated with correctly recalled categories in the cued condition, then a further 20 control adjectives would be added to the TMR set (total = 60). Inclusion of the control adjectives enabled a direct comparison of brain activity during cued memory reactivation and non-specific, matched auditory stimulation.

The mean ± SEM number of cues assigned to each condition were as follows. Nap group: 12.59 ± 0.73 object cues, 13.37 ± 0.82 scene cues, 13.63 ± 0.80 control stimuli. Wake group: 11.74 ± 0.91 object cues, 12.84 ± 0.74 scene cues, 12.53 ± 0.78 control stimuli. However, cues were presented continuously throughout the offline period (i.e., during late non-REM sleep in the nap group), so the mean ± SEM absolute number of cue presentations were as follows. Nap group: 69.44 ± 10.13 object cues, 74.48 ± 11.91 scene cues, 73.82 ± 11.16 control stimuli. Wake group: 97.26 ± 5.08 object cues, 108.63 ± 6.10 scene cues, 104.84 ± 4.62 control stimuli. Numbers of absolute cue presentations were applied to a 3 (Cue Type: Object/Scene/Control) x 2 (Group: Nap/Wake) mixed ANOVA. Because T1 category recall was numerically greater for scenes than objects, there were more scene than object cues assigned to the TMR set (Cue Type main effect [Huynh-Feldt corrected]: $F(1.07,46.94) = 6.94$, $p = 0.01$). In general, there was more cueing time available in the wake delay than in the nap delay, meaning that the wake group received more cues than the nap group (Group main effect: $F(1,44) = 5.09$, $p = 0.03$). Despite this difference, the wake group failed to exhibit any behavioral benefit of cueing, further demonstrating that TMR is – in the current paradigm - only effective at bolstering memory retention when delivered in sleep. There was no Cue Type*Group interaction ([Huynh-Feldt corrected]: $F(1.07,46.94) = 0.97$, $p = 0.34$). After EEG artifact rejection in the nap group, the corresponding numbers were: 67.63 ± 9.82 object cues, 71.74 ± 11.35 scene cues, 71.30 ± 10.65 control stimuli. There was no significant difference in the number of artifact-rejected cues in each condition ([Huynh-Feldt corrected] $F(1.14, 29.59 = 2.31$, $p = 0.14$).

Offline Period (Nap or Wakefulness)

The offline period began at ~2pm and lasted 90 min. Participants in the nap group were left to sleep in a laboratory bedroom while their brain activity was monitored with polysomnography (set up before the study began). TMR was initiated when participants were in late NREM stage N2/early stage N3. The TMR set was presented in a randomized order (ISI = 4 s ± 200 ms) at a sound intensity of ~40dB (as measured with a sound-level meter placed at the same position where participants laid their head on the pillow). After each full round of cueing, the adjectives were reshuffled and immediately presented again. Cueing continued for as long as participants were in sleep stage N2/N3, but immediately paused if they showed signs of micro-arousal or awakening, or moved into sleep stage N1 or rapid eye movement (REM) sleep. The cues were continued if participants re-entered sleep stage N2/N3 after an arousal.

Participants in the wake group played an online game (Bubble Shooter) for the first 30 min of the offline period. For the next 30 min, the TMR cues were presented continuously while participants completed a 1-back working memory task. This approach reduced the probability that participants directly attended to the cues during TMR [7, 9]. During the 1-back task, a series of random numbers between 0 and 10 were presented one after another in the center of the screen. The task was to indicate whether the current number was the same as or different to the number one digit prior. After completing the 1-back task, participants played Bubble Shooter again for the remaining 30 min of the offline period.

Follow-Up Tests (T2 and T3)

Participants returned 6 hours later for a follow-up test (T2). This followed the same procedures as T1 with the single exception that new foil adjectives were used. The next morning (after a night of sleep), participants completed another test (T3). Again, this followed the same procedures as T1 and T2, but with a new set of foils.

Discrimination Task

After completing T3, participants were informed of the true purpose of the study and asked if they had been aware of the auditory cues in the offline period. To assess their explicit knowledge of the cues, participants were asked to complete an adjective discrimination task. On each trial, one of 100 adjectives from the encoding phase was presented acoustically and visually displayed for 10 s. Participants were asked to indicate whether or not the adjective had been replayed during the offline period.

Equipment

Experimental Tasks and TMR

All experimental tasks and TMR algorithms were implemented on a PC with MATLAB 2015a and Psychtoolbox 3.0.13 [30]. In the wake group, adjective cues were presented via speakers connected to the task PC. In the nap group, cues were presented via a speaker mounted ~1.5 m above the bed, which was connected to an amplifier in a separate control room.

Polysomnography

An Embla N7000 PSG system with RemLogic 3.4 software was used to monitor sleep. After the scalp was cleaned with NuPrep exfoliating agent (Weave and Company), gold plated electrodes were attached using EC2 electrode cream (Grass Technologies). EEG scalp electrodes were attached according to the international 10-20 system at 8 locations: frontal (F3, F4), central (C3, C4), parietal (P3, P4) and occipital (O1, O2), and each was referenced to an electrode on the contralateral mastoid (A1 or A2). Left and right electrooculography electrodes were attached, as were electromyography electrodes at the mentalis and submentalis bilaterally, and a ground electrode was attached to the forehead. Each electrode had a connection impedance of < 5 kΩ. All online signals were unfiltered and digitally sampled at 200 Hz. Sleep scoring was carried out in accordance with the criteria of the American Academy of Sleep Medicine [35].

QUANTIFICATION AND STATISTICAL ANALYSIS

Data Analysis

Behavioral Data Analysis

Category recall was our primary measure of memory accuracy. We calculated for each participant: 1) the proportion of target categories recalled at T1 that were subsequently recalled at T2, and 2) the proportion of target categories recalled at T2 that were subsequently recalled at T3 (i.e., following a night of sleep). To avoid any ambiguity related to category memory, we excluded from our analyses any item that was incorrectly classified during the object/scene categorisation task. Across all participants, we excluded 162 items out of a possible 4600 (3.52%). Category recall scores at T1, T2 and T3 were normally distributed in both the nap and wake groups (Kolmogorov-Smirnov test, $p > .05$), and thus met the assumptions of analysis of variance (ANOVA). As such, the data were subjected to a 2 (TMR: Cued/Not-Cued) X 2 (Group: Nap/Wake) mixed ANOVA. The statistical significance threshold was set at $p < .05$. Behavioral data were analyzed with SPSS statistics 24.

EEG Data Analysis

EEG data were analyzed with MATLAB, using the FieldTrip [31] (v.06/02/2017) and CircStat [32] (v.1) toolboxes. The continuous sleep data were segmented into epochs from −1 s to 3 s around cue onset and subjected to a two-step artifact rejection procedure. In the first step, artifacts were automatically detected and removed based on the median ± 3.5 inter-quartile ranges of both signal amplitude and gradients (the difference between two adjacent samples) of all epochs. In the second step, the remaining epochs were manually screened via FieldTrip's visual summary functions and epochs containing amplitude, variance or kurtosis outliers were additionally removed. For TMR-cue-locked analysis of event-related potentials (ERPs), data were high-pass filtered at 0.5 Hz and baseline-corrected with respect to the −200 ms to 0 ms window before cue onset. For time-frequency representations (TFRs), data were convolved with a 5-cycles hanning taper and spectral power was obtained from 4-30 Hz in 0.5 Hz frequency steps and 5 ms time steps. For analyses, participant-specific TFRs were converted into percent power change relative to a −300 ms to −100 ms pre-cue window. Because our TFR analysis relied on extended data windows to fit 5 cycles per frequency (e.g., 15 Hz x 5 cycles = 333 ms), a −300 ms to −100 ms baseline window was chosen to mitigate baseline contamination by post-stimulus activity while preserving proximity to cue onset. Note though that TFR comparison of old cues versus control stimuli (Figure 3) revealed the same significant 13-16 Hz power increase when the TFR baseline window matched that of the ERP analysis (−200 to 0 ms).

For representational similarity analysis (RSA) of within- versus between-category processing, a sliding window of 200 ms (in steps of 10 ms) was applied to the 0.5 Hz high-pass filtered raw EEG data to obtain, for each trial, a series of 8-channel-by-41-time points

(200 Hz/5 ms sampling rate) EEG feature vectors [36]. Using these feature vectors, Spearman correlations were then used to quantify, for each time point, the representational similarity across all pairwise combinations of trials, resulting in an n trials x n trials correlation matrix. This matrix is symmetrical around the diagonal, and all cells below the diagonal as well as the diagonal itself were removed. Additionally, same-adjective correlations across multiple cueing rounds were removed (that is, we excluded correlations between e.g., adjective x, cueing round 1 and adjective x, cueing round 2). Next, within-category similarity was obtained by averaging across all remaining object-object and scene-scene cells. Between-category similarity was obtained by averaging across all object-scene cells. The numbers of within-category and between-category cells were equated by randomly sub-selecting cells from the majority class in each participant. Each participant's within-category and between-category correlation time series were Fisher z-transformed to adjust for non-normality of correlation coefficients.

All ERP, TFR and RSA analyses were performed as random-effects analyses (paired-samples t tests) and corrected for multiple comparisons using FieldTrip's nonparametric cluster-based permutation method (1000 randomizations), including channel x time (ERP), channel x time x frequency (TFR) and time (RSA) as cluster-defining features. The statistical significance threshold was set at $p < .05$.

EEG Event Detection

Sleep data were partitioned according to the time (minutes) spent in each stage of sleep (N1, N2, N3 and REM sleep). Data scored as N2 or N3 were extracted from all EEG channels for spindle and slow oscillation analysis. For spindles, data were first bandpass filtered from 10-13 Hz (slow spindles) or 13-16 Hz (fast spindles) using a 4th order two-pass Butterworth filter. We focus on 13-16 Hz because the significant cluster resulting from contrasting old cues versus control stimuli starts at 13 Hz (Figure 3B). Although that cluster slightly leaks into higher frequencies up to 19 Hz, we set 16 Hz as the upper limit to conform to the more conventional 12-16 Hz band for fast spindles [37]. Next, we took the envelope of the resulting signal and determined an amplitude threshold as mean + 1.25 SD. A spindle was then defined as an event that surpassed that threshold for a minimum of 0.5 s and a maximum of 3 s. For SO detection, data were filtered from 0.5-2 Hz using a 4th order two-pass Butterworth filter. Next, zeros crossings were detected in the resulting signal and events with two successive positive-to-negative crossings spanning 0.8-2 s were taken forward to the next step. Here, the resulting candidate events' trough and trough-to-peak amplitudes were calculated and events surpassing mean + 1.25 SD of both these metrics were considered SOs. In both event detection procedures, automatically detected artifact samples (see above) were padded for ± 1 s and those samples were excluded prior to event detection.

SO-Spindle Coupling

For determining the preferred phase of SO-spindle modulation, we first identified spindles whose maximum occurred from 1.5-2.5 s after onset of old memory cues (encompassing the interval in which we observed the spindle increase, Figure 3B). We then extracted a ± 1.5 s raw data segment around the spindle maximum (accommodating the maximum spindle duration of 3 s) and created one signal by filtering the data between 0.5 and 2 Hz and another signal by filtering the data between 13 and 16 Hz. For the lower frequency signal, instantaneous phase was extracted via the Hilbert transform. For the higher frequency signal, phase of the power envelope was extracted, again using the Hilbert transform. For each sample (601 samples, i.e., 3 s at 200 Hz sampling rate), the circular distance between the two phase time series was calculated and the mean resulting angle ('preferred phase') determined. For instance, if the spindle amplitude were to systematically peak at the SO down state (trough), the preferred phase would be 180°. Conversely, if the spindle amplitude was to – as hypothesized - systematically peak at the SO up state (peak), the preferred phase would be 0° (see also [25, 38]). Each participant's preferred phase of SO-spindle modulation was obtained from averaging all individual events' preferred phases, and the resulting distribution across participants was then tested against uniformity (Rayleigh test) as well as against uniformity with an *a priori* defined mean direction (V test).

DATA AND SOFTWARE AVAILABILITY

EEG and behavioral data are available upon request by contracting the Lead Contact, Bernhard Staresina (b.staresina@bham.ac.uk).

Current Biology, Volume *28*

Supplemental Information

Memory Consolidation Is Linked to Spindle-Mediated Information Processing during Sleep

Scott A. Cairney, Anna á Váli Guttesen, Nicole El Marj, and Bernhard P. Staresina

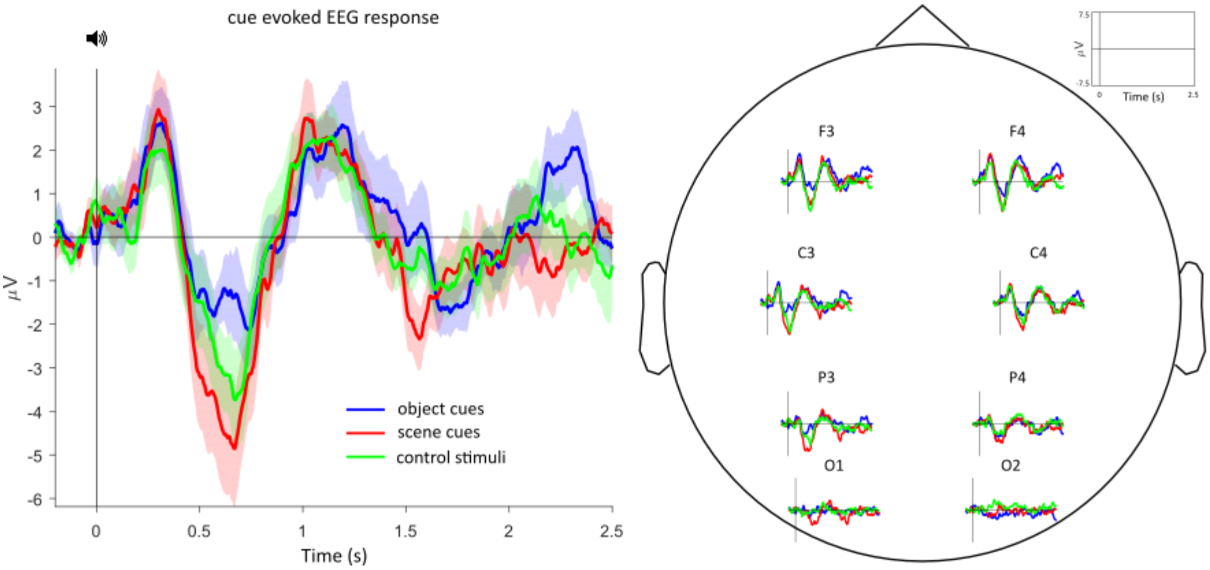

<u>Figure S1: Event Related Potentials (ERPs) in Response to Auditory Cues, Related to Figure 2B</u> *Left*: Traces show mean ± SEM across participants, collapsed across all electrodes. *Right*: Condition means across participants, shown for each electrode separately. A repeated-measures ANOVA revealed no significant condition effects comparing object cues, scene cues and control stimuli after controlling for multiple comparisons across time and electrodes.

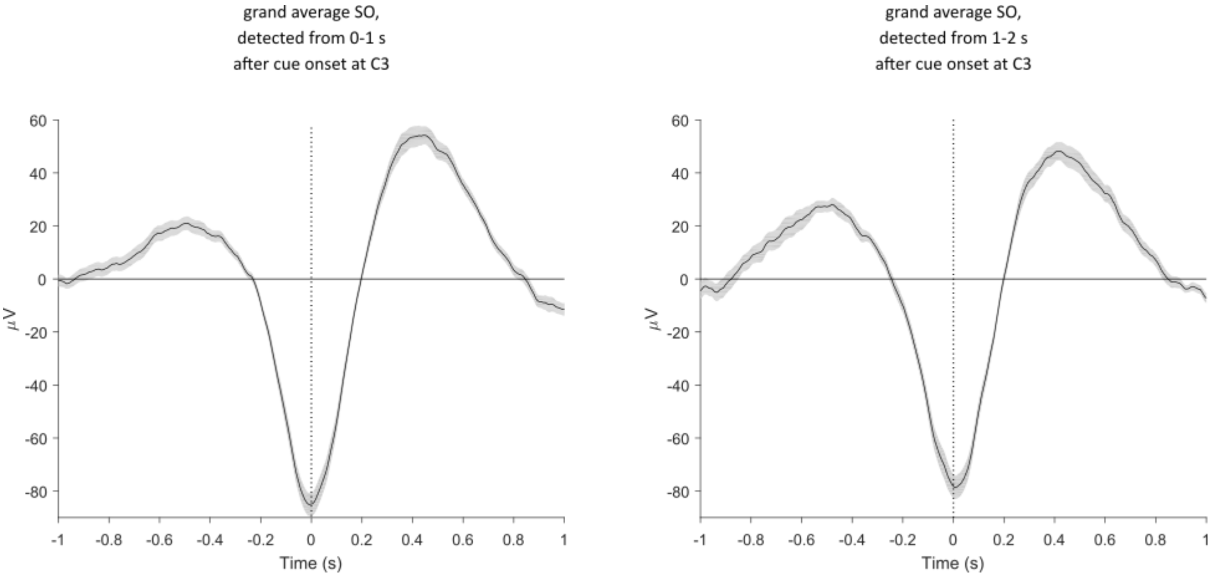

<u>Figure S2: Grand Average Slow Oscillations, Related to Figures 2 and 3.</u> Algorithmically detected slow oscillations (see STAR Methods) whose maximum trough (‘down state’) fell between 0-1 s (*left*) or 1-2 s (*right*) post cue onset, shown for electrode C3. Traces show grand average ± SEM across participants. ± 1 s raw data segments around the SO trough (time 0) were extracted to accommodate the maximum SO duration of 2 s. Note that both early and late SOs surpass the standard peak-to-peak amplitude criterion of 75µV [S2].

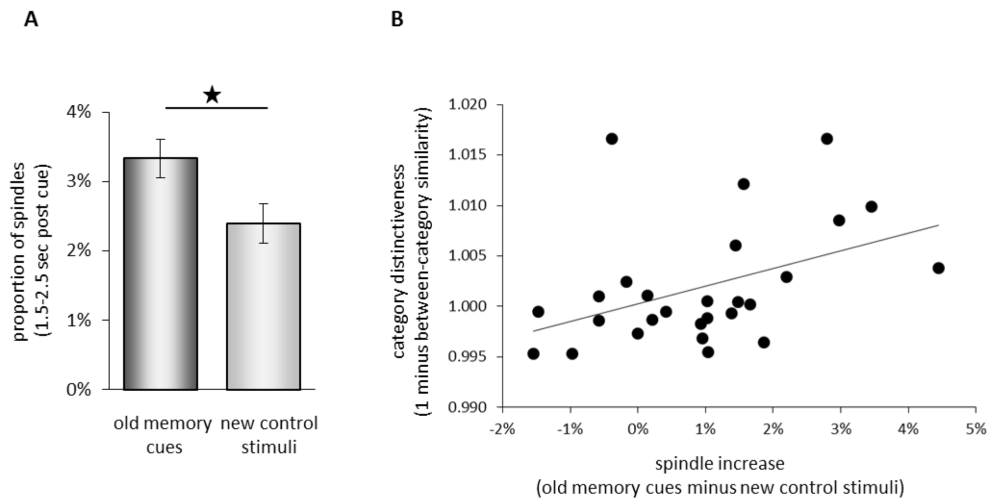

<u>**Figure S3: Discrete Spindle Events from 1.5-2.5 s post cue onset, Related to Figure 3**</u> **A.** Relative count of algorithmically detected fast spindles (13-16 Hz) for old memory cues and new control stimuli, averaged across left hemisphere electrodes. Star indicates significant difference ($t(26)=3.33$, $P=.003$) **B.** Across-participant correlation between (i) relative spindle increase for old cues vs. control stimuli (cf. panel **A**) and (ii) category distinctiveness of object- and scene-related cues (Spearman's rho=.47, $P=.014$).

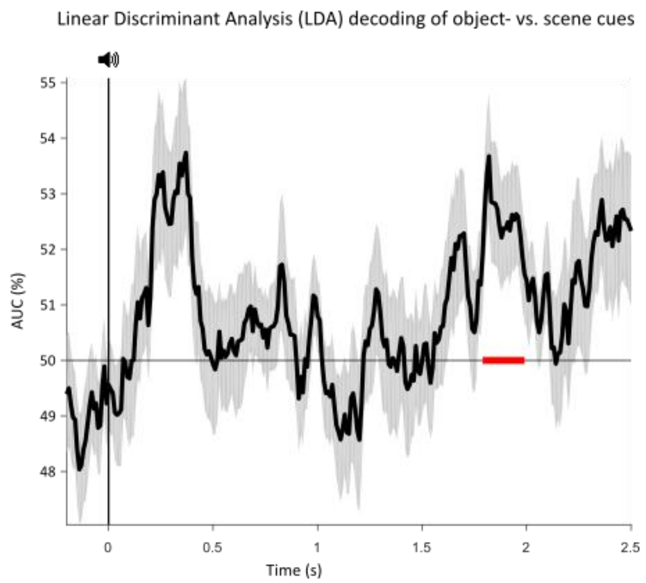

<u>**Figure S4: Linear Discriminant Analysis (LDA) decoding of object vs. scene cues, related to Figure 4A**</u> Classification was performed analogously to the RSA analysis described in the main text. At each timepoint (-.2 s to 2.5 s relative to cue onset, 10 ms steps), k-fold cross-validation was performed on an 8 channels x 41 timepoints (200 ms) feature vector. K was set to 5 and 5 repetitions were included to provide stability across different partitions of training and testing trials. Unequal trial numbers for object- vs. scene-related cues were handled by oversampling the minority class, i.e. a random sub-selection of trials from the smaller category was duplicated to match the number of trials in the larger category. Critically, this was only done during training and not during testing, avoiding any bias in cross-validation but using the whole range of data. The outcome measure was % area under the curve (AUC) of a classification Receiver Operating Characteristic (ROC), shown to be more sensitive than simple accuracy measures [S3, S4]. Chance classification would be 50%. Each participant thus provides a single cue-locked classification time course. Second-level significance was tested via FieldTrip's cluster-based permutation method (correcting for multiple comparisons across time), where initial clusters were obtained by one-tailed t-test of classification exceeding chance levels. Results show significant above-chance classification from 1.79 s to 1.99 s post cue onset ($P<.05$, corrected; red line), replicating the RSA

effects shown in Figure 4A. Note that classification is also significantly above chance when using the exact time window of significant RSA effects (1.76s-2.06s, $t(26)=2.9$, $P=.004$, one-tailed).

	T1 (%)				
	Hit	*Miss*	*CR*	*FA*	*d'*
Nap	84.04<br>(± 2.10)	15.96<br>(± 2.10)	96.37<br>(± 0.69)	3.63<br>(± 0.69)	2.89<br>(± 0.10)
Wake	89.74<br>± (1.71)	10.26<br>± (1.71)	96.42<br>(± 0.77)	3.58<br>(± 0.77)	3.15<br>(± 0.14)
	T2 (%)				
	Hit	*Miss*	*CR*	*FA*	*d'*
Nap	77.70<br>(± 2.54)	22.30<br>(± 2.54)	96.67<br>(± 0.72)	3.33<br>(± 0.72)	2.67<br>(± 0.11)
Wake	79.32<br>(± 3.27)	20.68<br>(± 3.27)	94.95<br>(± 1.98)	5.05<br>(± 1.98)	2.78<br>(± 0.16)
	T3 (%)				
	Hit	*Miss*	*CR*	*FA*	*d'*
Nap	79.74<br>(± 2.86)	20.26<br>(± 2.86)	96.52<br>(± 0.87)	3.48<br>(± 0.87)	2.83<br>(± 0.10)
Wake	80.11<br>(± 3.38)	19.89<br>(± 3.38)	94.95<br>(± 2.46)	5.05<br>(± 2.46)	2.78<br>(± 0.11)

Table S1: Adjective Recognition, Related to Behavioural Results The data (means ± SEM) refer to hits, misses, correct rejections (CR) and false alarms (FA) at each test phase. The sensitivity index (*d'*) [Normalized (hits/(hits + misses)) – Normalized (false alarms/(false alarms + correct rejections))] was calculated for adjective recognition memory. We adopted a log-linear approach to safeguard this analysis against errors arising from 0 and 1 values: for each participant, 0.5 was added to the total hits and total false alarms, and 1 was added to the total signal (old) trials and total noise (new) trials [S1]. Adjective recognition scores (*d'*) were applied to a 3 (Test: T1/T2/T3) X 2 (Group: Nap/Wake) mixed ANOVA. Recognition performance initially declined between T1 and T2, but then stabilised between T2 and T3 (Test main effect [Huynh-Feldt corrected]: $F(1.78,78.41)=9.00$, $P=.001$). However, these changes in performance were not modulated by the type of offline activity (Group*Session interaction [Huynh-Feldt corrected]: $F(1.78,78.41)=2.36$, $P=.11$). There was also no main effect of Group ($F(1,44)=0.53$, $P=.47$), indicating that overall adjective recognition performance was equivalent in the nap and wake groups.

All	*T1 (%)*	*T2 (%)**		*T3 (%)***	
		Not Cued	*Cued*	*Not Cued*	*Cued*
Nap	50.15 (± 2.97)	79.57 (± 2.14)	80.69 (± 2.45)	88.48 (± 1.58)	94.36 (± 0.84)
Wake	46.84 (± 2.89)	64.17 (± 4.10)	64.75 (± 3.66)	82.52 (± 3.61)	82.76 (± 3.14)

Objects	*T1 (%)*	*T2 (%)**		*T3 (%)***	
		Not Cued	*Cued*	*Not Cued*	*Cued*
Nap	48.96 (± 2.92)	80.86 (± 2.36)	80.20 (± 3.16)	90.09 (± 2.03)	96.14 (± 1.76)
Wake	47.37 (± 3.51)	67.47 (± 4.51)	68.01 (± 4.32)	84.54 (± 4.02)	88.72 (± 3.55)

Scenes	*T1 (%)*	*T2 (%)**		*T3 (%)***	
		Not Cued	*Cued*	*Not Cued*	*Cued*
Nap	51.33 (± 3.18)	78.14 (± 2.70)	80.78 (± 2.69)	87.77 (± 1.85)	92.27 (± 1.50)
Wake	46.32 (± 2.77)	61.40 (± 4.57)	61.61 (± 4.53)	79.14 (± 4.90)	76.14 (± 5.62)

Table S2: Category Recall, Related to Behavioural Results Data (means ± SEM) are shown for all items (top) and then separated for object and scenes. *Refers to the proportion (%) of T1-recalled categories that were also recalled at T2. **Refers to the proportion (%) of T2-recalled categories that were also recalled at T3. Note: 50% accuracy is not to be mistaken as chance performance given that participants had a "?" response option (see Figure 1A). We repeated the analyses reported in the main text to examine objects and scenes separately. A 2 (Type: Object/Scene) X 2 (Group: Nap/Wake) mixed ANOVA showed that object and scene recall did not differ at T1 (Type main effect: $F(1,44)=0.23$, $P=.64$), for either the nap group or the wake group (Type*Group interaction: $F(1,44)=1.54$, $P=.22$). Again, there was no main effect of Group ($F(1,44)=0.59$, $P=.45$). At T3, a main effect of Type $F(1,44)=6.88$, $P=.01$) indicated that objects were generally better recalled than scenes. However, there was no interaction between Type and any other factor(s) (all $P>.05$), suggesting that the memory effects of sleep and TMR observed in this study did not vary according to category membership. Objects were also better recalled than scenes at T2 (Type main effect: $F(1,44)=4.11$, $P=.05$). Again, there was no interaction between Type and any other factor(s) (all $P>.05$).

All	*T1 (%)*	*T2 (%)**		*T3 (%)***	
		Not Cued	*Cued*	*Not Cued*	*Cued*
Nap	83.48<br>(± 2.10)	84.93<br>(± 1.98)	84.54<br>(± 2.01)	94.06<br>(± 1.11)	96.43<br>(± 0.66)
Wake	77.35<br>(± 4.21)	68.45<br>(± 4.18)	70.74<br>(± 4.20)	89.17<br>(± 3.94)	87.52<br>(± 2.58)

Objects	*T1 (%)*	*T2 (%)**		*T3 (%)***	
		Not Cued	*Cued*	*Not Cued*	*Cued*
Nap	86.01<br>(± 2.29)	87.16<br>(± 2.41)	85.16<br>(± 2.78)	94.31<br>(± 1.64)	98.32<br>(± 0.82)
Wake	79.71<br>(± 4.32)	73.36<br>(± 4.85)	73.72<br>(± 4.89)	91.21<br>(± 3.61)	94.96<br>(± 2.39)

Scenes	*T1 (%)*	*T2 (%)**		*T3 (%)***	
		Not Cued	*Cued*	*Not Cued*	*Cued*
Nap	81.02<br>(± 2.45)	82.48<br>(± 2.61)	82.90<br>(± 2.22)	93.66<br>(± 1.43)	94.53<br>(± 1.33)
Wake	75.09<br>(± 4.44)	64.51<br>(± 5.05)	68.38<br>(± 5.15)	84.97<br>(± 5.61)	76.40<br>(± 7.22)

Table S3: Exemplar Recall, Related to Behavioural Results Data (means ± SEM) are shown for all items (top) and then separated for object and scenes. *Refers to the proportion (%) of T1-recalled items that were also recalled at T2. **Refers to the proportion (%) of T2-recalled items that were also recalled at T3. Note: the exemplar recall analysis was restricted to trials for which the category (object or scene) was correctly retrieved. At T1, more object exemplars were recalled than scene exemplars (Type (Object/Scene) main effect: $F(1,44)=8.92$, $P=.005$). There was no main effect of Group (Nap/Wake) ($F(1,44)=1.99$, $P=.17$) and no Type*Group Interaction ($F(1,44)=0.01$, $P=.91$). At T2, the nap group strongly outperformed the wake group (Group main effect: $F(1,44)=18.46$, $P<.0001$) and objects were better recalled than scenes (Type main effect: $F(1,44)=7.37$, $P=.009$). There was no main effect of TMR ($F(1,44)=0.07$, $P=.80$) and no interactions between factors ($P>.05$). At T3, there was again a main effect of Group ($F(1,44)=7.90$, $P=.007$) and a main effect of Type ($F(1,44)=9.64$, $P=.003$). There was also a Type*Group Interaction ($F(1,44)=4.67$, $P=.04$): objects were better recalled than scenes in the wake group but not in the nap group. While the TMR*Group interaction did not reach significance ($F(1,44)=2.57$, $P=.12$) in this analysis, planned t-tests revealed a strong trend towards superior recall for cued relative to non-cued items in the nap group ($t(26)=2.01$, $P=.06$, two-tailed), but not in the wake group ($t(18)=0.48$, $P=.64$). Taken together with our other findings, these results suggest that category level recall is most sensitive to the memory-enhancing effects of TMR. There were no other interactions between factors ($P>.05$).

**A**	*N1*	*N2*	*N3*	*REM*	*TST*
	14.52 (± 1.38)	35.78 (± 3.09)	14.85 (± 2.32)	20.70 (±2.84)	90.89 (± 2.47)

**B**	*Overall*	*F3*	*F4*	*C3*	*C4*	*P3*	*P4*	*O1*	*O2*
Slow Spindle Density	2.00 (± 0.10)	2.80 (± 0.18)	2.74 (± 0.17)	2.12 (± 0.14)	2.04 (± 0.14)	1.66 (± 0.09)	1.59 (± 0.10)	1.52 (± 0.06)	1.50 (± 0.06)
Fast Spindle density	3.37 (± 0.09)	3.10 (± 0.10)	3.04 (± 0.12)	3.70 (± 0.09)	3.45 (± 0.14)	3.92 (± 0.10)	3.66 (± 0.17)	2.99 (± 0.16)	3.12 (± 0.14)
SO *Density*	2.69 (± 0.08)	2.63 (± 0.11)	2.68 (± 0.10)	2.60 (± 0.09)	2.64 (± 0.08)	2.64 (± 0.10)	2.65 (± 0.08)	2.82 (± 0.08)	2.88 (± 0.07)

Table S4: Sleep EEG data, Related to EEG Results **A.** Time (min) spent in each stage of sleep and total sleep time (TST). **B.** Event density measures for slow spindles (10-13 Hz), fast spindles (13-16 Hz) and slow oscillations (SOs, 0.5-2 Hz) at each EEG channel. Density is calculated as the number of electrophysiological events divided by time (min) spent in non-rapid eye movement (NREM) sleep stages N2 and N3. Data are shown as means ± SEM.

Supplemental References

S1. Hautus, M.J. (1995). Corrections for extreme proportions and their biasing effects on estimated values of d'. Behavior Research Methods, Instruments & Computers 27, 46-51.

S2. Iber, C., Ancoli-Israel, S., Chesson, A., and Quan, S.F. (2007). The AASM Manual for the Scoring of Sleep and Associated Events: Rules, Terminology and Technical Specification. (Westchester (IL): American Academy of Sleep Medicine).

S3. Bradley, A.P. (1997). The Use of the Area Under the ROC Curve in the Evaluation of Machine Learning Algorithms. Pattern Recognition 30, 1145-1159.

S4. Rissman, J., Greely, H.T., and Wagner, A.D. (2010). Detecting individual memories through the neural decoding of memory states and past experience. Proceedings of the National Academy of Sciences 107, 9849-9854.
